# Rapid and Extensive Alteration of Phosphorus Speciation during Oxic Storage of Wet Sediment Samples

**DOI:** 10.1371/journal.pone.0096859

**Published:** 2014-05-06

**Authors:** Peter Kraal, Caroline P. Slomp

**Affiliations:** Department of Earth Sciences – Geochemistry, Faculty of Geosciences, Utrecht University, Utrecht, The Netherlands; University of Yamanashi, Japan

## Abstract

The chemical forms of phosphorus (P) in sediments are routinely measured in studies of P in modern and ancient marine environments. However, samples for such analyses are often exposed to atmospheric oxygen during storage and handling. Recent work suggests that long-term exposure of pyrite-bearing sediments can lead to a decline in apatite P and an increase in ferric Fe-bound P. Here, we report on alterations in P speciation in reducing modern Baltic Sea sediments that we deliberately exposed to atmospheric oxygen for a period of either one week or one year. During oxidation of the sediment, extensive changes occurred in all measured P reservoirs. Exchangeable P all but disappeared during the first week of exposure, likely reflecting adsorption of porewater PO_4_ by Fe(III) (oxyhydr)oxides (i.e. ferric Fe-bound P formation). Detrital and organic P were also rapidly affected: decreases in both reservoirs were already observed after the first week of exposure to atmospheric oxygen. This was likely because of acidic dissolution of detrital apatite and oxidation of organic matter, respectively. These processes produced dissolved PO_4_ that was then scavenged by Fe(III) (oxyhydr)oxides. Interestingly, P in authigenic calcium phosphates (i.e. apatite: authigenic Ca-P) remained unaffected after the first week of exposure, which we attributed to the shielding effect of microfossils in which authigenic Ca-P occurs in Baltic Sea sediments. This effect was transient; a marked decrease in the authigenic Ca-P pool was observed in the sediments after one year of exposure to oxygen. In summary, we show that handling and storage of wet sediments under oxic conditions can lead to rapid and extensive alteration of the original sediment P speciation.

## Introduction

There exists a suite of geochemical characterizations that are used to reconstruct the conditions under which marine sediments have been deposited, both in modern and ancient marine systems. Of particular interest is the redox state at the sediment-water interface during sediment deposition, as this greatly affects the mobility and cycling of essential nutrients and metals. Phosphorus (P) speciation can be a valuable tool, because the chemical distribution of P in marine sediments is strongly dependent on the redox state of bottom water and sediment. The popular ‘SEDEX’ chemical sequential extraction procedure, developed by Ruttenberg [1], allows separation of five important sedimentary P reservoirs: exchangeable P, ferric Fe-bound P, authigenic and biogenic calcium phosphates (Ca-P), detrital P, and organic P. The ferric Fe oxide-bound P pool forms through scavenging of dissolved phosphate (PO_4_) by Fe(III) (oxyhydr)oxides and can be an important P reservoir in oxic surface sediments. In contrast, ferric Fe-bound P is generally insignificant or absent in reducing sediments where Fe(III) (oxyhydr)oxides cannot form or persist. In addition, the ratio between organic carbon (C) and (organic) P can be used as an indicator of the redox conditions at the sediment-water interface, because organic C is more efficiently buried than (organic) P under oxygen-depleted conditions [2]. As such, P speciation holds valuable information on redox conditions during sediment deposition and early burial. Eventually, most labile P phases are converted into apatite (Ca-P), which is the principal long-term sediment P reservoir [3], [4].

However, careful sample handling is required to preserve the original geochemical sediment characteristics, including P speciation. Two recent studies provided evidence for alteration of the chemical P distribution in marine sediments during storage in contact with atmospheric oxygen. Lukkari et al. [5] reacted reduced surface sediments from the Baltic Sea with oxygen by mixing for 30 min. under air, and subsequently monitored P speciation over a period of three months, using a sequential extraction procedure that largely targets the same key P phases as SEDEX. The authors observed a ∼25% decrease in apatite P over three months that was closely matched by a concurrent increase in P associated with reducible Mn(IV) or Fe(III) phases. In addition, Kraal et al. [6] determined P speciation with the SEDEX procedure in ancient sediments that were deposited under strongly reducing conditions, but were stored in opened cores in contact with atmospheric oxygen for years or even decades. These sedimentary records showed remarkably high ferric Fe-bound P contents (up to 99% of total P) in combination with low authigenic Ca-P concentrations in carbonate-poor sediment intervals that were deposited under strongly reducing conditions and contained abundant pyrite. The authors explained these findings by redistribution of solid-phase P driven by pyrite oxidation, which is a well-known source of acidity and secondary oxidized Fe minerals in exposed pyritic soils and sediments [7]–[10]. First, apatite is dissolved by H_2_SO_4_ that is produced by pyrite oxidation. The released P is subsequently scavenged by Fe (oxyhydr)oxides that form when ferrous Fe (the other pyrite oxidation product) is oxidized and precipitates once it diffuses away from localized acidic regions where pyrite oxidation is occurring. The potential for rapid dissolution of apatite has been demonstrated in oxygenated pyrite/rock phosphate slurries that were inoculated with an iron-oxidizing bacterium, where up to 12% of apatite was dissolved within 84 hours [11].

While the potential for alteration of P speciation upon oxidation of sediment or soil has been shown, its rate and extent in relation to storage practice are not well understood. This is of great importance, as it is common practice to store sediments without effective shielding from oxygen for extended periods of time. For instance, sediment cores collected by the International Ocean Discovery Program (IODP) and its predecessors are cut lengthwise and opened, after which core halves are stored in contact with atmospheric oxygen prior to sampling. Here, we report on an experiment in which we exposed reducing carbonate-poor sediments from the Baltic Sea to the atmosphere for up to one year, and assessed the degree to which the sediment P speciation was altered compared to that in pristine samples that were handled under strictly anoxic conditions. The conditions were optimal for oxygen exposure and sediment oxidation, as the sample containers were regularly aerated and the sediment was kept at constant moisture at room temperature. Our results confirm that exposure to the atmosphere can rapidly and extensively alter sediment P speciation, and show that this includes effects beyond the conversion of apatite P to Fe(III)-bound P.

## Materials and Methods

For the sediment oxidation experiment, we used samples from the Bornholm Basin in the southern Baltic Sea (site BY5) that were collected during a cruise with RV Skagerak in 2008 as described in detail by Mort et al. [12]. The sediment at this site consists of dark-colored, fine-grained material that is carbonate-poor (<2 wt%) and rich in sulfur (>1 wt%) and organic C (>4 wt%). Bottom waters at the site are seasonally hypoxic and occasionally euxinic [13]. Previous work has shown that sediments from this locality contain abundant FeS and FeS_2_ [14].

The sediment core was sliced under strictly anoxic conditions in a nitrogen-filled glovebox directly after core collection. Wet sediment samples were kept frozen under an N_2_ atmosphere until further analysis. For this study, four 0.5–1 g wet subsamples from each of five well-mixed sediment samples from depth intervals between 5.5 and 26 cm were transferred into 15 mL centrifuge tubes. Subsampling was performed in an argon-purged anoxic glovebox. One set of subsamples was used to determine the gravimetric moisture content from weight loss after freeze-drying. The moisture contents were subsequently used to determine the dry sediment weights for all other wet subsamples. The second set of wet subsamples was immediately subjected to the ‘SEDEX’ sequential P extraction procedure developed by Ruttenberg et al. [1] as modified by Slomp et al. [15], but including the extraction step for exchangeable P. The SEDEX procedure (Table 1) sequentially extracts: (i) exchangeable P; (ii) ferric Fe-bound P; (iii) P in authigenic and biogenic calcium phosphate minerals; (iv) P in detrital minerals; (iv) P in organic matter. For the fresh wet subsamples, the first two steps of the SEDEX procedure (exchangeable P and ferric Fe-bound P) were carried out in a glovebox to avoid sample oxidation. The remaining subsamples were brought into contact with the atmosphere during storage at room temperature, shielded from direct sunlight. One set of subsamples was aerated daily for a week, while moisture content was maintained by daily measurements of the evaporative weight loss and adjusting for this with ultrapure H_2_O. The last set of subsamples was stored for a period of one year, during which aeration and moisture contents adjustments were performed intermittently. Moisture loss never exceeded 50% of the original moisture content. Storage of sediments without shielding from oxygen is fairly common, with the aforementioned oxic core storage practices of the IODP as a good example. A noteworthy difference is the storage temperature (room temperature for the investigated samples, 4 °C for IODP cores), which may decrease the rate at which oxidative sediment alteration occurs. The two sets of exposed subsamples were subjected to the SEDEX procedure after one week and one year of exposure, respectively. The latter sample set was analyzed in duplicate. Phosphorus concentrations in all extracts were measured by colorimetry (molybdenum blue; [16]), except for the dithionite solutions that were analyzed by inductively coupled plasma – optical emission spectrometry (ICP-OES). The abundance of the different P pools are presented as a percentage of the sum of the extracted P phases.

**Table 1:** Overview of the sequential phosphorus fractionation procedure used in this study.

Step	Extractant, extraction time	Target phase
1	1 mol L^−1^ MgCl_2_, 30 min	Exchangeable P
2*	25 g L^−1^ Na dithionite (buffered to pH 7.3 with Na citrate/Na bicarbonate), 8 h	Ferric Fe-bound P
3*	1 mol L^−1^ sodium acetate solution (buffered to pH 4 with acetic acid), 6 h	P in authigenic and biogenic Ca-P (‘authigenic Ca-P’)
4	1 mol L^−1^ HCl, 24 h	Detrital P
5	ash at 550°C (2 h), then 1 mol L^−1^ HCl, 24 h	Organic P

*These steps were followed by a wash step with 1 mol L^−1^ MgCl_2_. The P extracted in this wash step was added to the total P extracted during that step.

## Results

**Figure 1:**
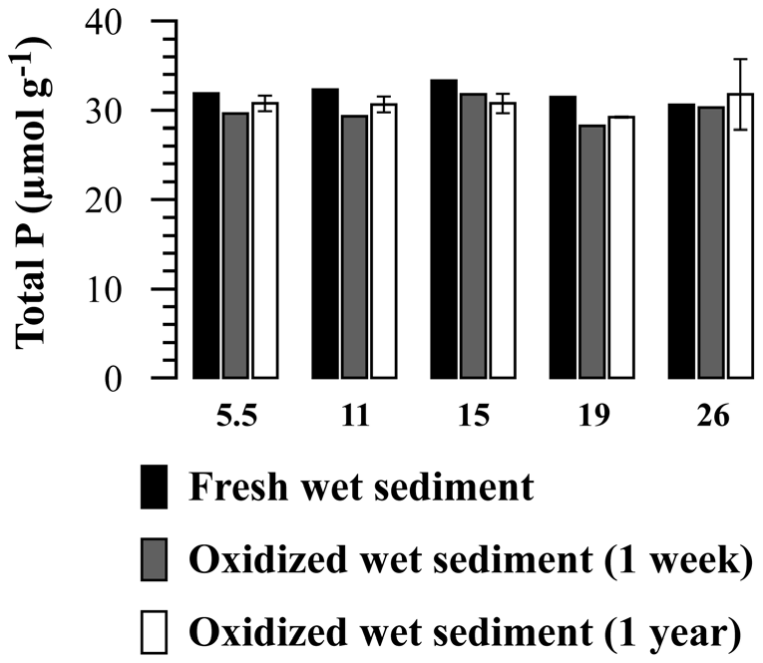
Total solid-phase phosphorus in the fresh Baltic Sea samples, and after one week or one year of exposure to atmospheric oxygen. Numbers on the x-axis represent the depth intervals (in cm) from which the samples were taken. Error bars for the samples that were exposed for 1 year represent the standard deviation over duplicate analyses.

**Figure 2:**
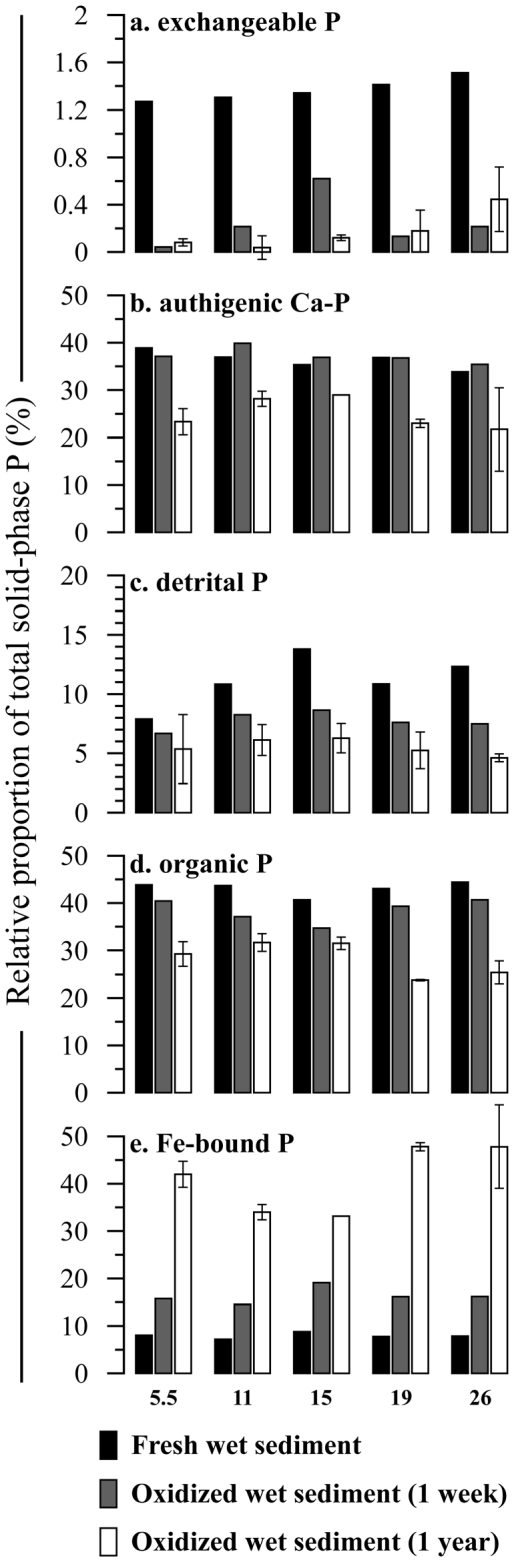
Phosphorus fractionation in fresh Baltic Sea samples, and after one week or one year of exposure to atmospheric oxygen. Phosphorus fractions are expressed as percentage of total SEDEX-extracted P. Numbers on the x-axis represent the depth intervals (in cm) from which the samples were taken. Error bars for the samples that were exposed for 1 year represent the standard deviation over duplicate analyses.

**Figure 3:**
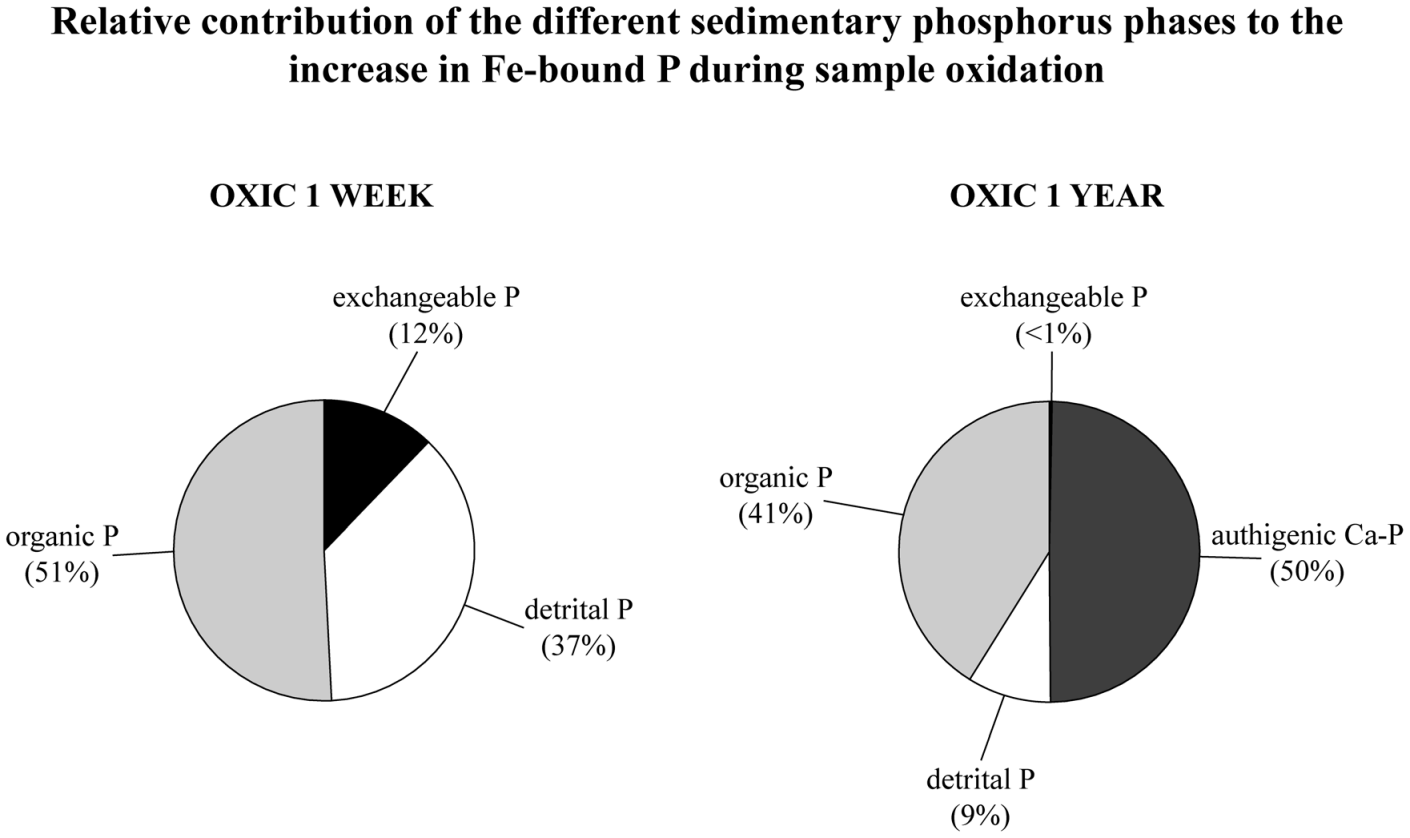
Relative contribution of the various P fractions to the increase of the ferric Fe-bound P fraction in sediments that were exposed to atmospheric oxygen for one week or one year. Numbers for each P phase are based on averaged values over the five samples; these were calculated for fresh and oxidized samples (one week or one year) and subsequently used to determine averaged changes in P speciation during sample oxidation.

The total size of the solid-phase P pool showed minor, non-systematic changes between the different storage conditions, demonstrating that changes in P fractionation were due to internal P conversions (Fig. 1). Phosphorus speciation in the five sediment samples was similar: on average, the P pool in the fresh samples consisted predominantly of organic P (43%) and authigenic Ca-P (36%), with minor contributions by detrital P (11%) and ferric Fe-bound P (8%) and a small pool of readily exchangeable P (1%). One week of exposure of wet sediment to atmospheric oxygen caused distinct changes in P speciation (Table 2; Fig. 2). Exchangeable P became nearly depleted and significant decreases were observed for detrital and organic P, while the authigenic Ca-P pool showed no systematic change. At the same time, a marked increase in the Fe-associated P pool was observed. After one year, exchangeable P remained negligible while further decreases in detrital and organic P were observed (Table 2; Fig. 2). In addition, the authigenic Ca-P pool showed a marked decline after one year. After the one-year oxidation period, ferric Fe-bound P was the largest P pool (41%) in the sediments, with decreased fractions of organic P (28%), authigenic Ca-P (25%) and detrital P (6%). Due to the differently timed responses of the individual P pools to sample oxidation, the main sources of ferric Fe-bound shifted from organic P (accounting for 51% of the increase in ferric Fe-bound P) and detrital P (37%) to authigenic Ca-P (50%) and organic P (41%) over the course of the exposure period (Fig. 3). Uncertainty as expressed by the error bars in Fig. 2 was likely caused by sample heterogeneity and subsequent, generally small, differences in oxidation effects between replicate sub samples.

**Table 2:** Changes in the abundance of phosphorus species after one week and one year of exposure to atmospheric oxygen, compared to fresh samples.

Sample	Oxic	exch. P		authi. Ca-P		detrital P		organic P		Fe-bound P	
5.5	fresh	0.01		0.39		0.08		0.44		0.08	
(32)	week	0.00	(−97%)	0.37	(−5%)	0.07	(−15%)	0.40	(−8%)	0.16	(96%)
	year	0.00	(−94%)	0.23	(−40%)	0.05	(−32%)	0.29	(−33%)	0.42	(423%)
11	fresh	0.01		0.37		0.11		0.44		0.07	
(32)	week	0.00	(−84%)	0.40	(8%)	0.08	(−24%)	0.37	(−15%)	0.15	(102%)
	year	0.00	(−97%)	0.28	(−24%)	0.06	(−44%)	0.32	(−27%)	0.34	(372%)
15	fresh	0.01		0.35		0.14		0.41		0.09	
(33)	week	0.01	(−54%)	0.37	(4%)	0.09	(−37%)	0.35	(−15%)	0.19	(117%)
	year	0.00	(−91%)	0.29	(−18%)	0.06	(−55%)	0.32	(−23%)	0.33	(277%)
19	fresh	0.01		0.37		0.11		0.43		0.08	
(32)	week	0.01	(−91%)	0.37	(0%)	0.08	(−30%)	0.39	(−9%)	0.16	(107%)
	year	0.00	(−87%)	0.23	(−38%)	0.05	(−52%)	0.24	(−45%)	0.48	(515%)
26	fresh	0.02		0.34		0.12		0.44		0.08	
(31)	week	0.00	(−86%)	0.35	(5%)	0.07	(−39%)	0.41	(−8%)	0.16	(105%)
	year	0.00	(−71%)	0.22	(−36%)	0.05	(−63%)	0.25	(−43%)	0.48	(506%)

Phosphorus pools given as fraction of total P, with the relative change in each P reservoir after one week and one year of exposure between parentheses. The absolute total P concentration for each sample, in µmol g^−1^, is given between parentheses in the first column.

## Discussion

Our results show that exposure of reducing sediment samples to atmospheric oxygen can lead to rapid and comprehensive alteration of sediment P speciation. In addition to the conversion of Ca-P to Fe-P observed in previous studies [5], [6], all measured P reservoirs (exchangeable P, authigenic Ca-P, detrital P, and organic P) were (partially) tranformed into ferric Fe-bound P upon sample oxidation. These alterations can overwrite the original P speciation that reflected depositional conditions, potentially rendering trends in P distribution meaningless for (paleo)environmental reconstruction. The conditions under which the samples in this study were exposed to oxygen, i.e. small sediment volumes that were regularly aerated at room temperature, represents an optimal scenario for sediment alteration through oxidation. The alterations described in this study likely proceed slower under cooled conditions, as is common practice for core storage (e.g. IODP core repositories). However, results by Kraal et al. [6] already indicated that long-term cooled storage under air of IODP sediment cores leads to marked alterations in sediment geochemistry. Importantly, the alteration of P speciation is strongly coupled to the oxidation of reduced Fe sulfide minerals, which are also commonly used to as indicators of (paleo)depositional conditions, thus further extending the impact of oxidative alteration of the original geochemical sediment characteristics. Here, we will highlight the impact of sample oxidation on the various P reservoirs, many of which are commonly used as proxies of geochemical conditions in the sediment.

Exchangeable P is usually a minor P reservoir in marine sediments and typically includes PO_4_ in pore water and P that is loosely bound to Fe-oxides [17]. In our samples, ‘exchangeable’ P amounted to only 0.4–0.5 µmol g^−1^. At a moisture content of around 70% as measured for these samples, ∼0.4 mL of porewater with a PO_4_ concentration of 200–400 µmol L^−1^ [12] in a wet sample of ∼0.5 g contributes ∼0.5 µmol P g^−1^ on a dry sediment weight basis. Thus, exchangeable P in our sediments can be attributed wholly to dissolved PO_4_ in the wet samples. The rapid removal of this PO_4_ pool with time can be explained by co-precipitation of the dissolved PO_4_ with Fe during formation of Fe(III) (oxyhydr)oxides upon sample oxidation [18].

The observed changes in the organic P pool show that oxidation of organic matter upon sample exposure also plays an important role in oxidation-driven changes to P fractionation, even on relatively short timescales. In fact, oxic decomposition of organic matter during the initial stages of sample oxidation was the most important source of P converted to ferric Fe-bound P (Fig. 3). Up to 45% of organic P was lost during the 1-year period of sample oxidation with oxygen (Table 2). This is in line with the observation that reactive organic matter that is preserved in sediments under anoxic conditions rapidly decays upon exposure to oxygen [19]. Irrespective of the overall composition (marine vs. terrestrial) of the organic matter in the Baltic Sea investigated here, the organic P pool undergoing change upon sample oxidation is most likely predominantly associated with the relative fresh marine organic matter that is most sensitive to oxygen. Initially, organic P was much more abundant than ferric Fe-bound P (43% and 8% of total P, respectively), as is common in reducing sediments. As a result, the conversion of a relatively small fraction of organic P (between 8% and 15%, Table 2) resulted in large changes in the ferric Fe-bound P pool. Apart from decreasing the overall size of the organic P pool, sample oxidation is likely to lead to selective preservation of relatively recalcitrant organic compounds and thus change the organic geochemical properties of the sample. Moreover, oxidation of labile organic compounds may alter the bulk organic C/organic P and organic C/total P ratios, which are used to reconstruct bottom water oxygenation [20]–[23].

Lastly, our results show that detrital P was affected by sample oxidation before authigenic Ca-P. Authigenic Ca-P, i.e. apatite extracted by a Na acetate/acetic acid solution buffered at pH 4, is generally considered to be more labile than detrital P, which consists of terrigeneous recalcitrant P minerals extracted by 1 M HCl (pH 0). Nonetheless, during the first week of the oxidation experiment detrital P decreased while authigenic Ca-P was more or less unaffected (Fig. 2b and c). This may be related to the occurrence of authigenic Ca-P in palynomorphs and other microfossils in Baltic Sea sediments [24]. Organic-walled microfossils and opal tests of dinoflagellates and diatoms, which are important phytoplankton species in the Baltic Sea [25], [26], can provide a protective niche that shields the apatite from dissolution by the sulfuric acid formed during pyrite oxidation. In contrast, detrital P likely occurs as terrigeneous particles scattered throughout the sediment; it is reasonable to assume that such unprotected detrital P is more sensitive to acidic dissolution than the authigenic Ca-P sequestered in microfossils. Physical protection in microfossils provides a cogent explanation for authigenic Ca-P preservation, yet currently unknown intrinsic differences in the size and chemical characteristics of authigenic Ca-P particles and detrital P-bearing particles may also underlie the different responses of these two sedimentary P pools to sample oxidation. No matter how the ‘protection’ of authigenic Ca-P was established exactly, the effect was relatively short-lived: in the period between one week and one year of sediment oxidation, dissolution of authigenic Ca-P was the main source of P for ferric Fe-bound P formation in the investigated sediments (Fig. 2 and 3). Although protection within microfossils may be effective in stored sediments, it does not prevent Ca-P dissolution during sequential extraction, when sediment samples are ground and then treated with an acidic solution involving constant agitation. The experimental conditions under which sequential extractions were performed greatly enhance the potential for dissolution of apatite by contact with the acidic solution.

Overall, we observed large changes in sedimentary P speciation in reducing Baltic Sea samples that were exposed to atmospheric oxygen, even after only one week. Sample oxidation does not significantly affect the total size of the solid-phase P reservoir, but does lead to the conversion of dissolved P, organic P, authigenic Ca-P and detrital P to ferric Fe-bound P. Sample oxidation thus greatly affects sediment P speciation (and the reduced character of the sediment in general) and obscures its relation to in situ depositional conditions. We recommend that samples intended for the study of P speciation are handled and stored under anoxic conditions. Given the impact of oxygen on P release from organic material, and in contrast to recommendations from previous work [6], this also holds for non-pyrite-bearing anoxic sediments. Appropriate storage can be achieved using gas-tight bags or jars flushed with nitrogen or argon. Storage at −20 C further limits the diffusion of oxygen and will halt the biogeochemical activity that leads to alteration of P speciation in stored sediments.
